# Long-term follow-up of *Helicobacter pylori* reinfection and its risk factors after initial eradication: a large-scale multicentre, prospective open cohort, observational study

**DOI:** 10.1080/22221751.2020.1737579

**Published:** 2020-03-11

**Authors:** Yong Xie, Conghua Song, Hong Cheng, Canxia Xu, Zhenyu Zhang, Jiangbin Wang, Lijuan Huo, Qin Du, Jianming Xu, Ye Chen, Xiaomei Zhang, Guoxin Zhang, Guibin Yang, Xiuli Zuo, Tao Guo, Yapi Lu, Fen Wang, Xuehong Wang, Kun Zhuang, Shiyao Chen, Wenzhong Liu, Nonghua Lu, on behalf of Chinese Society of Gastroenterology, Chinese Study Group on Helicobacter pylori and Peptic Ulcer

**Affiliations:** a Department of Gastroenterology, First Affiliated Hospital of Nanchang University, Nanchang, People’s Republic of China; b Department of Gastroenterology, Peking University First Hospital, Beijing, People’s Republic of China; c Department of Gastroenterology, 3rd Xiangya Hospital, Central South University, Changsha, People’s Republic of China; d Department of Gastroenterology, Nanjing First Hospital, Nanjing Medical University, Nanjing, People’s Republic of China; e Department of Gastroenterology, Sino-Japanese Friendship Hospital of Jilin University, Changchun, People’s Republic of China; f Department of Gastroenterology, First Affiliated Hospital of Shanxi Medical University, Taiyuan, People’s Republic of China; g Department of Gastroenterology, Second Affiliated Hospital of Zhejiang University College of Medicine, Hangzhou, People’s Republic of China; h Department of Gastroenterology, First Affiliated Hospital of Anhui Medical University, Hefei, People’s Republic of China; i Department of Gastroenterology, Nanfang Hospital, Southern Medical University, Guangzhou, People’s Republic of China; j Department of Gastroenterology, Hainan Hospital of Chinese People's Liberation Army General Hospital, Sanya, People’s Republic of China; k Department of Gastroenterology, First Affiliated Hospital of Nanjing Medical University, Nanjing, People’s Republic of China; l Department of Gastroenterology, Aerospace Center Hospital, Beijing, People’s Republic of China; m Department of Gastroenterology, Qilu Hospital of Shandong University, Jinan, People’s Republic of China; n Department of Gastroenterology, Peking Union Medical College Hospital, Beijing, People’s Republic of China; o Department of Gastroenterology, Zhongshan Hospital, Xiamen University, Xiamen, People’s Republic of China; p Department of Gastroenterology, Affiliated Hospital of Qinghai University, Xining, People’s Republic of China; q Department of Gastroenterology, Xi’an Central Hospital, Xi’an, People’s Republic of China; r Department of Gastroenterology, Zhongshan Hospital, Fudan University, Shanghai, People’s Republic of China; s Department of Gastroenterology, Renji Hospital, Shanghai Jiao Tong University School of Medicine, Shanghai, People’s Republic of China; t Department of Gastroenterology, Affiliated Hospital of Putian University, Putian, People’s Republic of China

**Keywords:** *Helicobacter pylori*, recurrence, reinfection, risk factor, epidemiology

## Abstract

*Helicobacter pylori* (*H. pylori*) recurrence remains a significant public health concern. The study aimed to assess *H. pylori* reinfection rate and identify its risk factors in China. This prospective open cohort, observational study was performed at 18 hospitals across 15 provinces in China. Consecutive patients who received the successful initial eradication during 1 January 2012 and 31 December 2018 were eligible for enrolment. *H. pylori* recurrence was defined as reinfection that occurred at more than the 12-month interval after successful initial eradication. Surveyed risk factors that might be associated with reinfection were preliminarily estimated by log-rank test and further determined by Cox regression model to calculate the hazard ratio (HR) and 95% confidence interval (CI). A total of 5193 subjects enrolled in the study. The follow-up intervals varied from 6 to 84 months with a general follow-up rate of 67.9%. Annual reinfection rate was 1.5% (95% CI: 1.2–1.8) per person-year. *H. pylori* reinfection was independently associated with the following five risk factors: minority groups (HR = 4.7, 95% CI: 1.6–13.9), the education at lower levels (HR = 1.7, 95% CI: 1.1–2.6), a family history of gastric cancer (HR = 9.9, 95% CI: 6.6–14.7), and the residence located in Western China (HR = 5.5, 95% CI: 2.6–11.5) following by in Central China (HR = 4.9, 95% CI: 3–8.1) (all *P* < 0.05). Reinfection rate of *H. pylori* in China is relatively low. Patients with specific properties of ethnic groups, education level, family history, or residence location appear to be at higher risk for reinfection.

## Introduction

*Helicobacter pylori* (*H. pylori*) is a Gram-negative bacterial pathogen that infects more than half of the human population worldwide [1]. The Kyoto global consensus report has defined *H. pylori* as a common source of infection [2], whose modes of transmission include oral-oral, faecal-oral and gastro-oral [3]. *H. pylori* is usually acquired during childhood and able to establish lifelong chronic infection [4]. Infected patients are asymptomatic in most cases but infection has been directly linked to chronic gastritis, peptic ulcer, non-ulcer dyspepsia, mucosa-associated lymphoid tissue lymphoma, and gastric cancer [5]. On the basis of compelling evidence, the World Health Organization has classified *H. pylori* as a group I carcinogen leading to gastric adenocarcinoma and recently highlighted the ranking of *H. pylori* in the priority list of research [6]. In addition, the extra-gastric manifestations also represent indeed one of the most fascinating and appealing issues of the whole history of *H. pylori* [7]. Therefore, Eradication of *H. pylori* is an effective strategy to prevent related gastrointestinal diseases and reduces the risk of relapse, such as peptic ulcer and gastric cancer [8].

At present, recommended treatment strategies usually comprise two antibiotics, a proton-pump inhibitor, and, in some regimens, bismuth salts [9]. These treatment protocols achieve eradication rates ∼90% [10]. To prevent progression of premalignant histological changes (such as atrophic gastritis and intestinal metaplasia) and recurrent peptic ulcer diseases, it is important to maintain *H. pylori* eradication status after successful treatment [11]. However, a negative result of a follow-up test after *H. pylori* treatment does not guarantee subsequent persistent eradication status in the future, even with the most effective treatment regimen currently available [12]. Studies suggest that the number of infected people has persisted or even increased over the past three decades because of *H. pylori* may be detected again after successful eradication [13]. Such a case is usually considered a recurrence. *H. pylori* recurrence also remains a significant public health concern in the management of *H. pylori* infection besides increasing antibiotic resistance [14].

The recurrence of *H. pylori* infection involves two distinct mechanisms: recrudescence and reinfection [15]. Recrudescence refers to the recurrence of the original strain of *H. pylori* that remains temporarily suppressed and undetectable posttreatment, whereas reinfection refers to infection by a new strain of *H. pylori* after successful eradication [16]. Obviously, the former is closely related to the failure of eradication treatment, while the latter is related to the transmission of infection again after successful eradication. Stewardship of *H. pylori* reinfection is more difficult than that of *H. pylori* recurrence, because the latter can be improved partially by adjusting the regimens. It is worth mentioning that the published rate and related factors of reinfection varied greatly among different countries depending on the survey region, population groups, sample size, socioeconomic status, research period, investigation methods, etc [13,17,18]. China has a vast territory and a large *H. pylor*-infected population that may exhibit complicated features of *H. pylori* reinfection. There has been no nationwide reinfection report of *H. pylori* in China till now, except for a few scattered regional studies [19–22]. The aim of the present nationwide survey was to assess the reinfection rate of *H. pylori* after successful initial eradication in China. In addition, we investigated the correlation between a series of potential risk factors and reinfection of *H. pylori*.

## Patients and methods

### Study design and protocol

This prospective open cohort, observational study was performed at 18 hospitals across 15 provinces in China. The potential risk factors that might be associated with the reinfection of *H. pylori* were determined by literature review and national conditions. To facilitate the collection and filling of concerned data, a structured case report form (CRF) was jointly designed by an expert panel, all of them are the members of the Chinese Study Group on *Helicobacter pylori* and Peptic Ulcer. The normative CRF mainly consisted of the following four sections: (1) Demographic information; (2) Socioeconomic status; (3) Individual behaviour; (4) Medical records. Each study centre used this uniform CRF, in which all formatted questions was required and standardized response options were provided. Because of the descriptive, exploratory nature of this observational study without statistical parameters, no pilot sample size and power calculations were performed.

### Study subjects

Consecutive patients with initial *H. pylori* infection were successfully eradicated in each study centre between 1 January 2012 and 31 December 2018 are eligible for enrolment. Moreover, all participants must also be in accordance with the following two criteria for enrolment and during the course of follow-up.

- Inclusion criteria:
  - Volunteering for participation;
  - Age between 18 and 65 years;
  - Absence of malignant disease, neuropsychiatric disorders or other serious chronic diseases;
  - Eradication regimens were limited to triple therapy, quadruple therapy (bismuth containing, sequential, concomitant, hybrid) or others recommended in the Maastricht III-V/Florence Consensus Report or Third-Fifth Chinese National Consensus Report on the management of *H. pylori* infection.
  - Cooperation to finish the detailed questions about the potential risk factors of *H. pylori* reinfection as required in designed CRF;
  - Agree to re-check the negative status of *H. pylori* again at 4 weeks after the initial confirmation of successful eradication;
  - Willing to comply with follow-up plan to reconfirm the status of *H. pylori* at specified interval which also stated by telephone notification in advance.
- Exclusion criteria:
  - Prepare for pregnancy or lactation duration;
  - A history of gastric surgery or received endoscopic therapy (e.g. endoscopic mucosal resection or endoscopic submucosal dissection);
  - Therapies containing other compounds such as probiotics, Chinese patent drugs or herbs, etc.;
  - Failure to understand correctly or express clearly during interviews;
  - Out of the study area or touch;
  - Oral or informal reconfirm report of *H. pylori* infection status.

### Follow-up schedule

All participants for each centre were interviewed by a well-trained interviewer to complete the designed questions and options regarding demographic information, socioeconomic status, individual behaviour, and medical records as required in CRF. The following specific variables were explored from each participant during the enrolment process:

- Demographic information: gender, ethnicity, age, educational level, marital status, the geographical location of permanent residence.
- Socioeconomic status: occupation class, the average monthly earnings, living environment, sanitation conditions, family size, per capita residential floor space (number of people sleeping in a house divided by the number of bedrooms).
- Individual behaviour: personal hygiene awareness, sharing the same glass, washing before eating, washing after defecation, tobacco use, drinking/alcohol intake;
- Medical records: a family history of gastric cancer, other family members infected with *H. pylori*, relevant diagnosis, eradication regimens, duration of regimens, types of proton pump inhibitors, combinations of antibiotics.

The follow-up interval to reconfirm the status of *H. pylori* was set as 6 months for the first time point after successful eradication of *H. pylori* infection and every 12 months thereafter. The criterion for terminating the follow-up was the recurrence of *H. pylori* infection at any point during the course of follow-up.

### Definitions

- Successful eradication: the initial eradication of *H. pylori* infection before follow-up schedule is defined as the achievement of negative *H. pylori* status at least 4 weeks after treatment in a previously *H. pylori*-infected patient. And the re-checking results at another 4 weeks after that will be deemed as a real negative-status of *H. pylori* after initial eradication treatment.
- Diagnostic criterion: the status of *H. pylori* infection during the follow-up period was directly determined by one or more of standard detection methods (limited to urea breath tests, histological staining, bacterial culture or faecal antigen testing). Diagnosis of *H. pylori* infection should meet the corresponding criteria in consensus. The performance of reconfirming within 4 weeks of receiving proton-pump inhibitors, H_2_ receptor antagonists, antibiotics, bismuth salts or endoscopy should be avoid. If this happens, it should be executed again at an appropriate time (at least 4 weeks later).
- *H. pylori* recurrence: *H. pylori* recurrence referred to the situation of *H. pylori* status became positive again in a patient with previously confirmed successful eradication. The recurrence of *H. pylori* was defined as a recrudescence that occurred during the 6–12 months period immediately after successful eradication. The recurrence of *H. pylori* was defined as reinfection that occurred more than 12 months after successful eradication.

### Review statement and informed consent

This observational study was free of charge, including the medical cost of re-examination of *H. pylori* during the follow-up period. The study protocol was reviewed and approved by the Chinese Medical Association & Chinese Society of Gastroenterology and the institutional review board of each participating centre. The objective of the study was explained to all patients before their participation, and written informed consent was obtained from all participants.

### Data processing and statistical analysis

The data of CRF were entered in a structure form with Epi Info^TM^ software for Windows (version 7.2, CDC, Atlanta, GA). The primary endpoint was recurrence cases. As the follow-up time markedly varies among different published studies, the risk of reinfection is better expressed as “yearly” infection. Therefore, the annual reinfection rate of *H. pylori* was calculated as percentage per person-year: [number of participants who became positive status of *H. pylori* during the defined period/sum of all participants during the observation years] × 100%.

Data of potential risk factors were presented as the mean ± standard deviation for continuous variables followed a normal distribution and as number (%) for categorical variables. Surveyed risk factors that might be associated with reinfection were preliminarily estimated by the log-rank test. Multivariable analyses with the Cox regression model was used to verify the independent predictors that influenced the reinfection events, in which the results were reported as hazard ratio (HR) and 95% confidence interval (CI). The Kaplan-Meier survival curve was used to depict events of the reinfection and remaining negative of *H. pylori* over time. The SPSS statistical software package for Windows (version 25.0, Inc., Chicago, IL, USA) was performed for all calculations. Differences were considered to be statistically significant when the *P*-value was 0.05 or less. All statistical tests were two-sided.

## Results

### Characteristics of participants in follow-up

**Figure 1.**
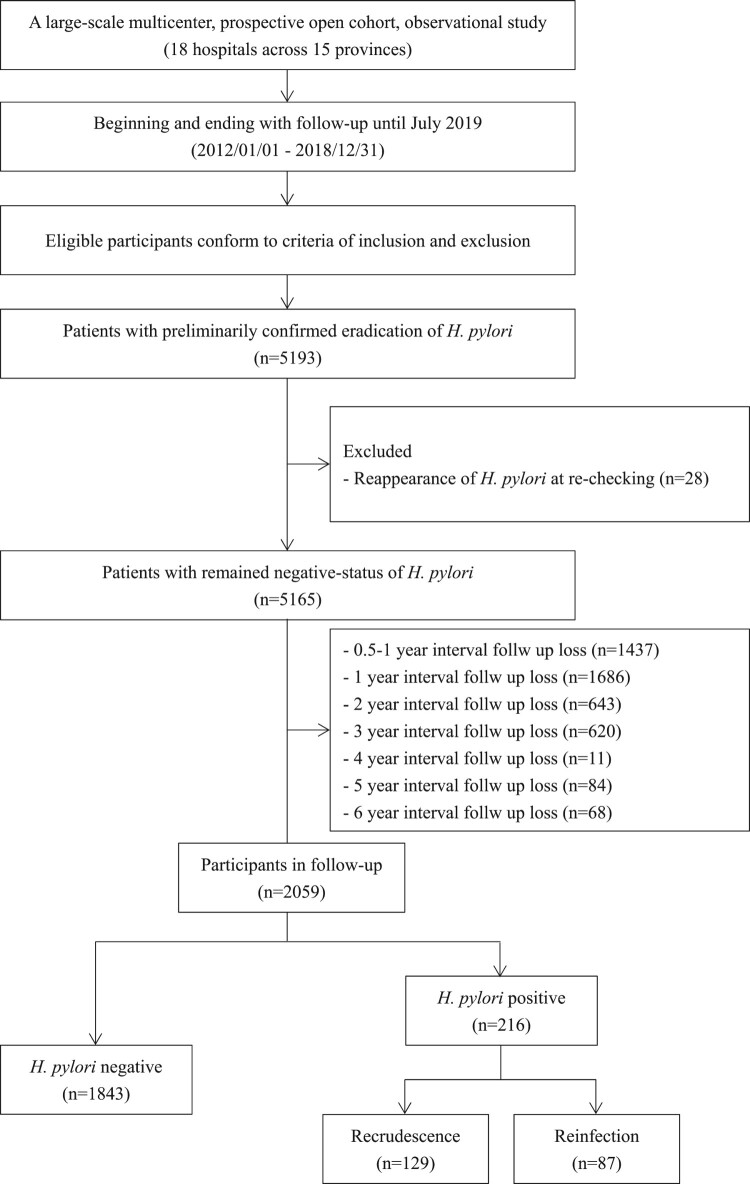
Study flow chart with criteria of inclusion and exclusion.

A total of 5193 subjects enrolled in a prospective open cohort during the enrolment process. However, 28 of these subjects were excluded from the further visit cohort for the reasons of *H. pylori* reappearance during re-checking at 8 weeks after successful eradication, and 1437 were excluded for the follow-up loss at first visit point. Therefore, a total of 3728 participants that remained negative status in a prospective open cohort were eligible for the follow-up. During the follow-up period, the number of participants who moved from the study area or were lost to follow-up at each visit interval was 1686, 643, 620, 11, 84, 11, and 68, respectively. Therefore, the overall follow-up rate in a prospective open cohort was 67.9% (range, 36.4–95.6%). Follow-up interval after successful eradication varied from 6 to 84 months and median follow-up duration was 58.2 ± 13.6 months. Ultimately, a total of 2059 participants in the prospective open cohort, with a median age of 47.3 ± 14.8 years, including 946 (46%) females and 1113 (54%) males, completed the follow-up at least once. A ﬂow diagram of the subject’s progress through the phases of the study is shown in Figure 1.

### *H. pylori* reinfection

During the follow-up, a total of 216 participants who experienced successful eradication of *H. pylori* in the prospective open cohort showed *H. pylori*-positive again (as recurrence cases). Among which, 129 participants showed as recrudescence cases of *H. pylori* infection at the 6-month interval after successful eradication, while 87 participants showed as reinfection cases of *H. pylori* infection at every 12-month interval thereafter. Because of a long follow-up period, the number of participants had decreased in each visit interval to certain extents. Based on the above number of participants we followed, a 1.5% per person-year (87/5707.5 person-year) (95%CI: 1.2–1.8) was calculated as the annual reinfection rate of *H. pylori*. The follow-up interval and the time when recurrences were found are summarized in Table 1.

**Table 1.** Annual reinfection rate of *Helicobacter pylori*.

Follow-up				Reinfection (*n*)	Person-years	Annual reinfection rate (%)
Time interval	Ideality (*n*)	Reality (*n*)	Rate (%)			
1≤year<2	1930	1287	66.7	21	1930.5	1.1
2≤year<3	1257	637	50.7	24	1592.5	1.5
3≤year<4	252	241	95.6	17	843.5	2
4≤year<5	224	140	62.5	9	630	1.4
5≤year<6	131	120	91.6	13	660	2
6≤year<7	107	39	36.4	3	253.5	1.2
Total	3728	2059	55.2	87	5910	1.5^a^

^a^There was no statistically significant difference in reinfection rate between each follow-up period (*P *= 0.842).

### Risk factors for the reinfection of *H. pylori*

**Figure 2.**
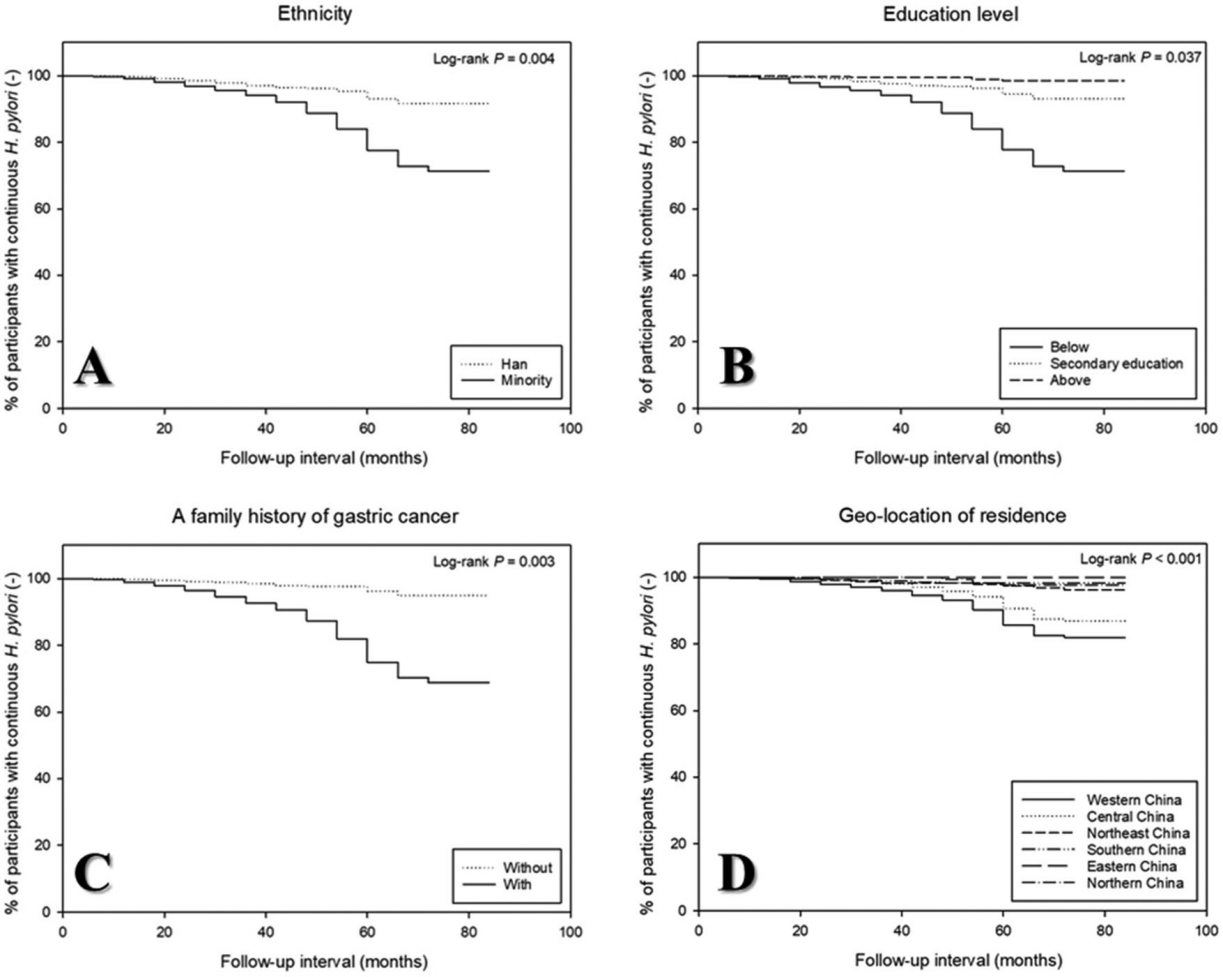
Kaplan–Meier curves for *Helicobacter pylori* reinfection according to ethnicity (A), educational level (B), a family history of gastric cancer (C), and geo-location of residence (D).

The potential risk factors affecting the recurrence of *H. pylori* infection were explored from each participant during the enrolment process, in which the survey had an overall response rate of ≥80%. Univariate analysis revealed that potential risk factors, including ethnicity, educational level, geo-location of residence, a family history of gastric cancer (all *P* < 0.1), suggesting that these factors possibly affected *H. pylori* infection recurrence after successful eradication therapy (Table 2). Further multivariate analysis showed that *H. pylori* reinfection was independently associated with the following five risk factors: minority groups (HR = 4.7, 95% CI: 1.6–13.9), the education at lower levels (HR = 1.7, 95% CI: 1.1–2.6), a family history of gastric cancer (HR = 9.9, 95% CI: 6.6–14.7), and the residence located in Western China (HR = 5.5, 95% CI: 2.6–11.5) following by in Central China (HR = 4.9, 95% CI: 3–8.1) (all *P* < 0.05), as shown in Table 3. Differences between the reinfection and non-reinfection groups were also observed when Kaplan-Meier curves were compared depending on these independent risk factors, and the curve of reinfection occurrence was depicted in Figure 2(A–D).

**Table 2.** Univariate analysis of risk factors associated with *Helicobacter pylori* reinfection.

Factors	Category	Total *N* (%)	Reinfection		*P*-value^a^
			Yes *n* (%)	No *n* (%)	
Total		2059 (100)	87 (100)	1972 (100)	
Gender		2059 (100)	87 (100)	1972 (100)	
	Male	1113 (54.1)	42 (3.8)	1071 (96.2)	0.269
	Female	946 (45.9)	45 (4.8)	901 (95.2)	
Ethnicity		2059 (100)	87 (100)	1972 (100)	
	Minority	21 (1)	4 (19.1)	17 (80.9)	0.004
	Han	2038 (99)	83 (4.1)	1955 (95.9)	
Age (years)		2059 (100)	87 (100)	1972 (100)	
	18–25	62 (3)	4 (6.5)	58 (93.5)	0.877
	26–35	414 (20.1)	16 (3.9)	398 (96.1)	
	36–45	550 (26.7)	24 (4.4)	526 (95.6)	
	46–55	682 (33.1)	30 (4.4)	652 (95.6)	
	56–65	298 (14.5)	10 (3.4)	288 (96.6)	
	66–70	53 (2.6)	3 (5.7)	50 (94.3)	
Educational level		2023 (98.3)	86 (98.9)	1937 (98.2)	
	Below	726 (35.9)	42 (5.8)	684 (94.2)	0.037
	Secondary education	906 (44.8)	30 (3.3)	876 (96.7)	
	Above	391 (19.3)	14 (3.6)	377 (96.4)	
Marital status		2010 (97.6)	85 (97.7)	1925 (97.6)	
	Yes	1902 (94.6)	78 (4.1)	1824 (95.9)	0.342
	No	108 (5.4)	7 (6.5)	101 (93.5)	
Occupational class		2031 (98.6)	82 (94.3)	1949 (98.8)	
	Unemployed	362 (17.8)	10 (2.8)	352 (97.2)	0.609
	Farmer	699 (34.4)	32 (4.6)	667 (95.4)	
	Worker	319 (15.7)	17 (5.3)	302 (94.7)	
	Merchant	267 (13.1)	10 (3.7)	257 (96.3)	
	Officer	204 (10)	8 (3.9)	196 (96.1)	
	Professional	180 (8.9)	5 (2.8)	175 (97.2)	
The average monthly earnings (RMB, ￥)		2059 (100)	87 (100)	1972 (100)	
	<1000	486 (23.6)	31 (6.4)	455 (93.6)	0.076
	1000–3000	959 (46.6)	33 (3.4)	926 (96.6)	
	>3000	614 (29.8)	23 (3.7)	591 (96.3)	
Geo-location of residence		2059 (100)	87 (100)	1972 (100)	
	Eastern China	1003 (48.7)	24 (2.4)	979 (97.6)	<0.001
	Western China	34 (1.7)	4 (11.8)	30 (88.2)	
	Southern China	173 (8.4)	9 (5.2)	164 (94.8)	
	Northern China	209 (10.2)	9 (4.3)	200 (95.7)	
	Northeast China	398 (19.3)	23 (5.8)	375 (94.2)	
	Central China	242 (11.8)	18 (7.4)	224 (92.6)	
Living environment		2037 (98.9)	87 (100)	1950 (98.9)	
	Urban	755 (37.1)	42 (5.6)	713 (94.4)	0.027
	Rural	1282 (62.9)	45 (3.5)	1237 (96.5)	
Sanitary level around residence		2011 (97.7)	85 (97.7)	1926 (97.7)	
	High	629 (31.3)	17 (2.7)	612 (97.3)	0.063
	Medium	814 (40.5)	42 (5.2)	772 (94.8)	
	Low	568 (28.2)	26 (4.6)	542 (95.4)	
Family size		2050 (99.6)	87 (100)	1963 (99.5)	
	1	21 (1.0)	1 (4.8)	20 (95.2)	0.267
	2	254 (12.4)	6 (2.4)	248 (97.6)	
	3–4	955 (46.6)	38 (4.0)	917 (96.0)	
	≥5	820 (40.0)	42 (5.1)	778 (94.9)	
Per capita living space (m^2^)		2031 (98.6)	86 (98.9)	1945 (98.6)	
	<10	138 (6.8)	5 (3.6)	133 (96.4)	0.159
	10–30	1130 (55.6)	58 (5.1)	1072 (94.9)	
	31–50	702 (34.6)	21 (3)	681 (97)	
	>50	61 (3.0)	2 (3.3)	59 (96.7)	
Personal hygiene awareness		2025 (98.3)	85 (97.7)	1940 (98.4)	
	High	316 (15.6)	9 (2.8)	307 (97.2)	0.196
	Medium	918 (45.3)	46 (5)	872 (95)	
	Low	791 (39.1)	30 (3.8)	761 (96.2)	
Sharing the same glass		2039 (99)	87 (100)	1952 (99)	
	Always	1209 (59.3)	45 (3.7)	1164 (96.3)	0.142
	Seldom	830 (40.7)	42 (5.1)	788 (94.9)	
Washing before eating		2050 (99.6)	86 (98.9)	1964 (99.6)	
	Always	1889 (92.6)	75 (4)	1814 (96)	0.125
	Seldom	161 (7.9)	11 (6.8)	150 (93.2)	
Washing after defecation		2050 (99.6)	84 (96.6)	1966 (99.7)	
	Always	1879 (91.7)	73 (3.9)	1806 (96.1)	0.159
	Seldom	171 (8.3)	11 (6.4)	160 (93.6)	
Tobacco use		2056 (99.9)	85 (97.7)	1971 (99.9)	
	Yes	1965 (95.6)	79 (4)	1886 (96)	0.349
	No	91 (4.4)	6 (6.6)	85 (93.4)	
Drinking/alcohol intake		2056 (99.9)	85 (97.7)	1971 (99.9)	
	Yes	1729 (84.1)	66 (3.8)	1663 (96.2)	0.131
	No	327 (15.9)	19 (5.8)	308 (94.2)	
A family history of gastric cancer		2018 (98)	86 (98.9)	1932 (98)	
	With	79 (3.9)	9 (11.4)	70 (88.6)	0.003
	Without	1939 (96.1)	77 (4)	1862 (96)	
A family member with *H. pylori*		2053 (99.7)	86 (98.9)	1967 (99.7)	
	Yes	327 (15.9)	18 (5.5)	309 (94.5)	0.252
	No	1726 (84.1)	68 (3.9)	1658 (96.1)	
Relevant diagnosis		2059 (100)	87 (100)	1972 (100)	
	Chronic gastritis	859 (41.7)	30 (3.5)	829 (96.5)	0.183
	Peptic ulcer	1034 (50.2)	52 (5)	982 (95)	
	Others	166 (8.1)	5 (3)	161 (97)	
Eradication regimens		2059 (100)	87 (100)	1972 (100)	
	Triple therapy	724 (35.2)	23 (3.2)	701 (96.8)	0.104
	Quadruple therapy	1335 (64.8)	64 (4.8)	1271 (95.2)	
Duration of regimens		2059 (100)	87 (100)	1972 (100)	
	7 d	691 (33.6)	28 (4.1)	663 (95.9)	0.165
	10 d	323 (15.7)	8 (2.5)	315 (97.5)	
	14 d	1045 (50.8)	51 (4.9)	994 (95.1)	
Types of proton pump inhibitors		2059 (100)	87 (100)	1972 (100)	
	Ilaprazole	25 (1.2)	0 (0)	25 (100)	0.119
	Rabeprazole	494 (24.0)	18 (3.6)	476 (96.4)	
	Lansoprazole	134 (6.5)	5 (3.7)	129 (96.3)	
	Pantoprazole	606 (29.4)	32 (5.3)	574 (94.7)	
	Omeprazole	164 (8.0)	2 (1.2)	162 (98.8)	
	Esomeprazole	536 (26.0)	30 (5.6)	506 (94.4)	
Combinations of antibiotics		2059 (100)	87 (100)	1972 (100)	
	Amoxicillin + Clarithromycin	650 (31.6)	23 (3.5)	627 (96.5)	0.296
	Amoxicillin + Furazolidone	1094 (53.1)	55 (5)	1039 (95)	
	Amoxicillin + Tetracycline	84 (4.1)	4 (4.8)	80 (95.2)	
	Amoxicillin + Metronidazole	68 (3.3)	0 (0)	68 (100)	
	Amoxicillin + Levofloxacin	102 (5)	3 (2.9)	99 (97.1)	
	Others	61 (3)	2 (3.3)	59 (96.7)	

^a^Log-rank test.

**Table 3.** Multivariate analysis of risk factors associated with *Helicobacter pylori* reinfection.

Risk factors	*P*^a^	HR	95% CI	
			Lower	Upper
*Geo-location of residence*				
Northern China	Reference			
Central China	<0.001	4.9	3.0	8.1
Northeastern China	0.052	1.9	1.0	3.8
Southern China	0.64	0.6	0.1	4.6
Western China	<0.001	5.5	2.6	11.5
Eastern China	0.578	1.9	0.2	19.0
*Ethnicity*				
Han	0.005	5	1.6	10
Minority				
*Education level*				
Above	Reference			
Secondary education	0.444	0.7	0.3	1.7
Below	0.027	1.7	1.1	2.6
*A family history of gastric cancer*				
With	<0.001	9.9	6.6	14.7
Without				

^a^Cox regression model.

## Discussion

Effective eradication of *H. pylori* is essential for the risk reduction of developing gastric cancer [23]. However, certain patients will infection again after successful eradication [13,24,25]. It remains a serious problem worldwide, especially in less-developed areas with a high prevalence of *H. pylori* infection or gastric cancer [26]. Because of the rate of *H. pylori* reinfection is low, perhaps diseases associated with the infection will also decline accordingly [27]. The major results of this prospective open cohort study performed at 18 hospitals across 15 provinces with a long-term follow-up (6–84 months) in China were that the annual reinfection rate was 1.5% (95% CI: 1.2–1.8) per person-year.

As outlined in a recent comprehensive meta-analysis covering 132 studies (53,934 person-year) from 45 countries or regions by Hu et al., the global annual reinfection rate of *H. pylori* was 3.1% (95% CI: 2–5), which remained relatively stable over the past few decades but varied across different regions [25]. In this study, it can be seen that the current annual reinfection rate in China is lower than the global level (1.5% vs. 3.1%). Similarly, we also found that the reinfection case could occur at each interval during follow-up and these differences were not statistically significant (*P *< 0.05).

There were reports that the incidence of *H. pylori* reinfection varies with regional development [28]. Gisbert et al [13] reviewed the annual recurrence rate after eradication was about 3.4% in developed countries and 8.7% in developing countries, respectively. The main reason for this difference is that recurrence of *H. pylori* usually has been considered to be due to recrudescence in developed countries. Whereas studies from developing countries suggest the main cause for recurrence of *H. pylori* is reinfection [16]. Distinguishing between reinfection and recrudescence can be a challenge in *H. pylori* infection without DNA fingerprints [29]. However, it is not easy to perform in clinical practice. Reports have shown that the recurrence rates of *H. pylori* decrease with time and decline sharply after the first year and come close to the rate of natural acquisition of *H. pylori* infection in adulthood [13,16,30]. Reinfection contributes to 62.5% of cases of *H. pylori* recurrence in the first 6 months after eradication, as well as most cases in the first year [31]. Furthermore, this definition is supported by data obtained using DNA analysis that the cause of *H. pylori* recurrence after the first year is reinfection [16].

In our study with large sample size, the re-checking results at 8 weeks after eradication treatment will be used as the real status of *H. pylori* infection to eliminate the false-negative cases (0.54%, 28/5193). Like the reasons mentioned above, we distinguished between recrudescence and reinfection in recurrence cases with a one-year interval after successful eradication. Therefore, our criteria to judge recurrence are consistent with their definitions that identical strains were detected in a significant proportion of *H. pylori* which became re-positive at early follow up (at 6–12 months), while *H. pylori* which became re-positive at later follow up (>1-year interval) were all different strains [32]. Therefore, the results of this study are comparable to published data from similar studies.

Recrudescence is most common when low-efficiency therapies are used, while reinfection requires re-exposure and is, therefore, more likely in countries with high *H. pylori* prevalence and poor sanitation [33]. As for the lower annual reinfection rate in China, it might be caused by the significant improvements in the socio-economic situation as well as health and living conditions over the past decades. Analogously, it may be not surprising that such an inconsistent result of the annual reinfection rate has emerged in Asia-Pacific countries. A long-term prospective study with 1609 patients followed for up to 12.5 years (mean 4.7 years) in Japan showed the reinfection rate of *H. pylori* was very low (0.22%) [27], while similar long-term follow-up study (18–95 months) in Korea showed the annual reinfection rate was 3.51% per year [34]. Moreover, the recurrence rate also varies (0–2.3%) among developed western countries or community (e.g. the United States, European Union and Australia) [25,35,36].

*H. pylori* recurrence rate is inversely correlated with its regional Human Development Index (HDI) and sanitation conditions [25,28]. Currently, following factors have been proposed as risk factor for reinfection, including age, dental plaque, close contacts, contaminated endoscopic equipment, eating habits, drinking water, low income, etc [37–44]. In our study, there are five risk factors: minority groups (HR = 4.7, 95% CI: 1.6–13.9), the education at lower levels (HR = 1.7, 95% CI: 1.1–2.6), a family history of gastric cancer (HR = 9.9, 95% CI: 6.6–14.7), and the residence located in Western China (HR = 5.5, 95% CI: 2.6–11.5) following by in Central China (HR = 4.9, 95% CI: 3–8.1), while other reported factors were not found to be the independent affecting factors in our study. Nevertheless, the findings of this study are generally consistent with the HDI. However, the underlying mechanism of its correlation needs further modelling analysis and in-depth clinical verification.

The present study still has some limitations. Firstly, DNA fingerprint technology is the golden standard for identifying the difference between recrudescence and reinfection. Secondly, despite efforts made by each centre to recruit all participants during the enrolment process, some participants did not complete a follow-up plan ideally. Therefore, we have a certain degree of computational bias in evaluating the rate of *H. pylori* reinfection. Thirdly, the re-treatment information was not available in the current medical records, and we could not evaluate the effectiveness of subsequent regimens in reinfection patients. That topic is what we will focus on in the future. Finally, because reinfection cases are much less than non-recurrence cases in practice, type I statistical errors are prone to be made in judging the relevant risk factors.

## Conclusion

In conclusion, long-term follow-up of the present open prospective cohort study shows that the annual reinfection rate (1.5% per person-year) of *H. pylori* after initial eradication in China is relatively low when compared with other developing countries. However, we still need to be wary of qualitative changes arising from quantitative translation. Patients with specific properties of residential location, ethnic groups, education level, or family history appear to be at higher risk for reinfection. Therefore, different strategic surveillance and appropriate reassessment should be taken to prevent the reinfection of *H. pylori* in high-risk populations.

## Supplementary Material

Supplemental Material
